# Supplementary material for: Highly Sensitive and Selective Detection of Hydrogen Using Pd-Coated SnO2 Nanorod Arrays for Breath-Analyzer Applications
Source: Sensors (Basel). 2022 Mar 7;22(5):2056. doi: 10.3390/s22052056 (PMC8914855; doi:10.3390/s22052056)
Supplement: Supplementary file 1 [file sensors-22-02056-s001.zip › sensors-1604881-supplementary.pdf]

Supplementary Material

Highly sensitive and selective detection of hydrogen using Pd-coated SnO_2 nanorod arrays for breath-analyzer applications

Hwaebong Jung[1], Junho Hwang[1], Yong-Sahm Choe[2], Hyun-Sook Lee[1,*], Wooyoung Lee[1,*]

[1] *Department of Materials Science and Engineering, Yonsei University, 50 Yonseiro, Seodaemun-gu, Seoul, 03722, Republic of Korea*
[2] Isenlab Inc., Halla Sigma Valley, Dunchon-daero 545, Jungwon-gu, Seongnam-si, Gyeonggi-do, 13215, Republic of Korea

* Corresponding author email: wooyoung@yonsei.ac.kr (W. Lee), h-slee@yonsei.ac.kr (H.-S. Lee)

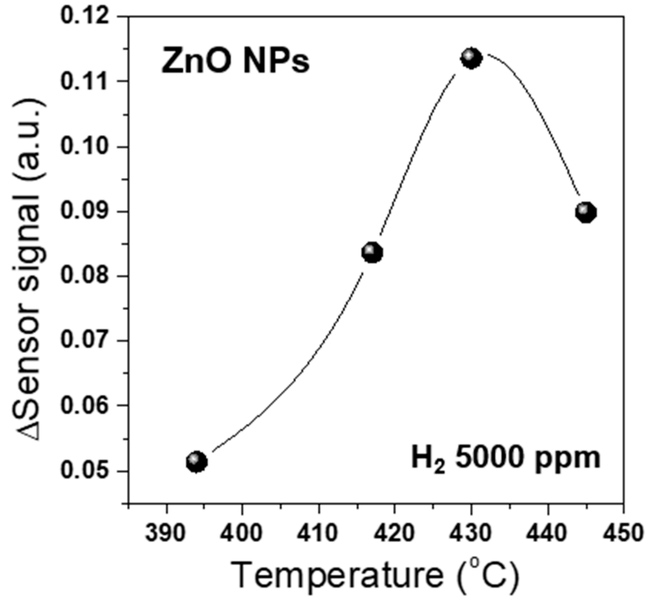

Figure S1. *Δ Sensor signal* of ZnO nanoparticles integrated into the mini-GC to 5000 ppm (0.5%) hydrogen as a function of operating temperature.
